# "The Hospital" Nursing Mirror

**Published:** 1897-08-28

**Authors:** 


### "The Hospital" Nursing Mirror.

Being the Nursing Section of "The Hospital."

[Contributions for this Section of "The Hospital" should be addressed to the Editor, The Hospital, 28 & 29, Southampton Street, Strand, London, W.C., and should have the word "Nursing" plainly written in left-hand top corner of the envelope.]

### News from the Nursing World.

### THE DUCHESS OF YORK AND NEEDLEWORK.

The Duchess of York takes great interest in the art of needlework. Some little time ago a cushion of her own work was exhibited in London, and it is, therefore, not a matter for surprise that Lady Cadogan's Textile Exhibition, and especially the stall of the Royal Irish School of Art Needlework, should attract her attention. Shortly after the Duchess's arrival at the Viceregal Lodge she sent for Miss Beresford, and after making many kindly inquiries about the society, ordered a white satin dress to be embroidered in the new French ribbon work, and a workbox, the fac-simile of one used by Marie Antoinette. This action on the part of the Duchess is sure to be popular, as the peasantry of Ireland are famed for the beauty of their needlework. Let us hope that it will also stimulate the interest of the public in the endeavours of such societies to provide employment for them, and thus help Ireland's women.

### A QUESTION OF COURTESY.

Some nurses object to address the medical man under whom they are working as "Sir," and they substitute the term "Doctor" for it. It has been stated that the ground for this objection is that many nurses are ladies by birth and education, and that they are thus not only the social equals of medical men, but in some cases their superiors. Therefore it is derogatory for them to say "Sir." This argument is wrong, firstly, because the question stands entirely apart from social position; and secondly, because if allowed to pass it would make an unfair distinction between nurses who have social advantages and those who have not. The title "Sir" is the appropriate and courteous mode, prescribed by custom, by which those acting under orders shall address those giving them; thus, the school-boy calls his master "Sir," the subaltern addresses his superior officer in this way, and in the etiquette of a bygone day children and young people used it in speaking to their elders. To object to saying "Sir" under the proper circumstances shows oneself ignorant of good manners. A lady entering the nursing profession as a means of earning her livelihood is neither more nor less than any other nurse. She must accept the position along with the duties and emoluments.

### PAUPER NURSING IN IRISH WORKHOUSES.

A deputation, consisting of Lord Mounteagle, K.P.; Sir Francis Cruise, M.D.; Sir Thornley Stoker, M.D.; Sir William Thompson, president Royal College of Surgeons; Katherine Maguire, M.D.; Dr. T. H. Moorhead, president of Irish Medical Association: Dr. Joseph Smyth; Major Everard, D.L.; Mr. Francis Sheridan, J.P.; Chairman Navan Board of Guardians; and Mr. Thomas Kennedy, hon. secretary Irish Workhouse Association, was received by the Local Government Board on the 13th inst. The proceedings were private, but it is generally understood that the conference, which related to the recent report on "Nursing in Workhouse Infirmaries and Fever Hospitals," was satisfactory, and that another step is taken in the direction of abolishing pauper nurses.

### THE VICTORIA NURSES' INSTITUTE, SOUTH AFRICA.

Mrs. Mary H. Buchanan, in making an appeal for further funds in order to complete the necessary arrangements for the Victoria Nurses' Institute, says that there is money to buy a house in Cape Town, and that as soon as it is ready the matron and nurses will take possession. Mr. Rhodes has given £50, in addition to his original gift of £25.

### "VICTORIA REGINA; OR, A JUBILEE DREAM."

Miss Beatrice Lucas-Shadwell has written a story entitled "Victoria Regina; or, a Jubilee Dream," which has met with some success in the local paper. She has just published it in book form, and the proceeds go to the East Sussex Hospital. The Queen and the Princess of Wales have each accepted a copy.

### FREE COPIES OF "NURSING NOTES."

The Editress of *Nursing Notes* asks us to state that only country midwives who are members of the Midwives' Institute, and who belong to a recognised institution such as Queen Victoria's Institute for Nurses, the Workhouse Nursing Association, &c., are eligible to receive free copies of the journal upon application with stamped wrappers. Owing to a misapprehension of the terms upon which it is supplied, a large number of midwives, who are not members of the Midwives' Institute, nor of any recognised association, have sent wrappers for copies to the office of *Nursing Notes*.

### THE REPORT OF THE NURSES' CO-OPERATION.

The Nurses' Co-operation has issued its sixth report. The address is 8, New Cavendish Street, *not* 6, as stated last week. The number of nurses on the list at the close of last year was 400, a number which has been considerably increased since. The gross receipts reached £33,395, of which £30,892 was paid to the nurses. After deducting working expenses, £1,006 was added to capital, which now, with donations and members' subscriptions, amounts to £3,067. Many members of the nursing staff are now members of the Royal National Pension Fund and are so entitled to sick pay. The committee will be glad when every member on the staff avail themselves of the privileges offered by the Pension Fund, and thus free themselves from all pecuniary anxiety on account of sickness and age. Miss Amy Hughes succeeds Miss Philippa Hicks as lady superintendent. She was trained at the Nightingale School, St. Thomas's Hospital, and has acted as superintendent of the Central Nursing Home, Q.V.J.I., and also as superintendent of nurses at Bolton Infirmary, where she introduced trained nurses.

### CARRICKFERGUS JUBILEE NURSING ASSOCIATION.

Miss Dunn, superintendent of the Queen Victoria Jubilee Institute for Nurses, Dublin, addressed a public meeting in the Albert Hall, Carrickfergus, setting forth the advantages to be obtained by affiliating the new nursing association with that of the Queen's Nurses. Miss Dunn divided her address under five heads—(1) The origin of district nursing; (2) of what it consisted, and how nurses were trained for it; (3) the class of people who needed it; (5) what affiliation with the Jubilee Institute meant. Mr. Daniel Bowman, J.P., occupied the chair; but the attendance was small on account of the bad weather.

### NUNS AS NURSES.

The rules by which nuns may become nurses of the sick in workhouses have been clearly defined by a recent letter of the Local Government Board to the clerk of the Portumna Union. If the Sisters from Ballinasloe give their services, the Guardians may accept them without the formality of electing them as nurses; but if it be proposed to pay them for their services, either the Guardians must be personally responsible for their salaries, or the Sisters must be duly appointed as Union officers in the manner prescribed by the general regulations.

### OVERWORK IN HULL INFIRMARY.

Too strict economy is a costly thing, as guardians find when they measure prosperity only by a pecuniary standard. Human life is seen to be precious when a young and good girl dies, partly, if not altogether, from overwork. It was the sad duty of the Hull Guardians, at a recent meeting of the board, to express their regret at the death of Nurse Longley. It was also recognised then that not only had she been overworked, but that the reason was the understaffing of the infirmary. It is a sharp lesson, but one not lost if the fault be remedied, as seems probable in this case.

### LLANDUDNO JUBILEE NURSE FUND.

The new Nurses' Institute is rapidly becoming organised. Lady Augusta Mostyn has been elected president, Mrs. Henry Mostyn vice-president, Mrs. T. T. Marks secretary, and Mrs. Wolseley Lewis treasurer. Some little time ago Mrs. Morgan raised £115, which is now in the bank and available for the fund, and about £70, mostly in the form of annual subscriptions, is also in hand. A nurse is to be appointed soon.

### WOMEN'S WORK IN INDIA.

An English corporal in India, writing to his people, says that the lack of home interests and companionship s one of the causes of the present trouble in the army. Could "homes" be made in every station, working as that in Rawalpindi, and the influence of such women as Miss Sandes and her helpers be brought to bear on the young soldiers, they would be most powerful instruments for good. This is essentially women's work, and with the model of a successful "soldiers' home" to copy, surely there are noble and capable women in India who will respond to the call.

### THE REPORT OF THE COLONIAL NURSING ASSOCIATION.

The Colonial Association is a year old, and the record of the year's work has proved all that its founders hoped for it. The aim of this association may fitly be called Imperial. It desires to link all British Colonies and Settlements together in one nursing association, of which the headquarters is London. A fair beginning has been made toward realising this aim. Three trained nurses have been sent to the Government hospital at Accra. The Colony of Lagos, which already had two English nurses at the Government hospital, sent home for a third, and nurses are at work now in Mauritius and Cyprus. A suitable candidate is being selected for St. Vincent, Dominica has applied for a midwife, and Sierra Leone has asked for two nurses, who will be sent out as soon as arrangements can be made. Influential residents in Bermuda, Newfoundland, the Straits Settlements, several protected native States, Nagasaki, and Yokohama are corresponding with the secretary now upon the subject, and are expected to apply shortly for nurses. The guarantee fund is only £500, and must be considerably augmented before other risks can be undertaken. It is desired to start a yearly subscribers' list, and efforts are being made to add to the number of persons interested in it. There are few residents in the British Isles who have not some dear ones in one or another quarter of the globe, and the thought that they may be sick in a distant land without a nurse ought to be a spur that should quicken their generosity towards a society whose successful work will supply the need.

### THE HOSPITAL STAFF AT JOHANNESBURG.

The Hospital Board at Johannesburg has recently established a training school for nurses in connection with the hospital. A number of probationers have been engaged on a three years' agreement, lectures will be given by the medical staff, and every facility will be offered to enable them to acquire a technical and practical knowledge of their profession. At the end of their training they will receive certificates. The board expects that this training school will be of great advantage to the hospital staff, and will in addition enable young women in the Transvaal to secure efficient training. Miss Young is the superintendent and Sister Adele the matron. The new arrangements have so far worked well. Sister Adele has held her present position since 1888, and her duties have been arduous. At one time, after the enlargement of the hospital, the nursing staff was inadequate, and 29 trained European nurses were engaged. They arrived in charge of Miss Young in January last. Two unfortunately died of fever shortly after, and several resigned for various reasons. The new training-school, which will enable the governing board to train its own nurses, will thus relieve the hospital authorities of a great responsibility and of much anxiety. At the same time it will lessen the demand for home trained nurses.

### SHORT ITEMS.

A sum of £33 was raised by means of a successful concert at Dunoon in aid of the Jubilee Nurse Fund.—A concert in the recreation-room of Glenburn Hydropathic Establishment realised £20 for the funds of the Rothesay Nursing Association. Another £20 was added by the cruise of the "Strathmore" to Lochgoil. About 400 were present in spite of bad weather. The "Strathmore" was lent for the occasion. — Queen Victoria Nursing Institution has received a cheque for £3 17s., the result of a local subscription in the parish of Slebech, Haverfordwest. A cheque for the same amount has been sent to the Prince of Wales's Hospital Fund. —About £1,900 of the Nottingham County Jubilee Fund falls to the share of the Nottingham Federation and District Nursing Association, and a proposal to invest the whole amount in the names of trustees was unanimously carried at a recent meeting.

### Lectures to Surgical Nurses.

By H. A. Latimer, M.D. (Dunelm), M.R.C.S. of Eng., L.S.A. of London, Consulting Surgeon, Swansea Hospital; President of the Swansea Medical Society; Lecturer and Examiner of the St. John Ambulance Association, &c.

### XI.—INFLAMMATORY FEVER—DESCRIPTION OF THE LYMPHATIC SYSTEM—ANALYSIS OF THE SYMPTOMS OF FEVER—TEMPERATURES, AND HOW TO ASCERTAIN THEM—VARIETIES OF SURGICAL FEVERS.

I purposely refrained in my last lecture from dealing with the constitutional symptoms resulting from inflammation, as there was not time enough then for properly discussing the matter. Foremost amongst the results of that condition is the one known as fever, under which name a good many phenomena are grouped. Before considering these phenomena, I must explain how they arise. When a part of the body is inflamed, poisons, resulting from decomposition of the fluids formed at the part, or derived from the germs which are the first cause of the disease, are absorbed by means of veins or of the lymphatic system, and from thence gaining access to the blood, set up the disturbance elsewhere, which results in fever. I must describe this lymphatic system before I go any further. Everywhere in the body are to be found little spaces, communicating with fine tubes, running from the extremities to the interior of the body. These tubes, or lymphatic vessels, here and there, and especially at the bends of the joints, run into little almond-shaped bodies, which are called lymphatic glands, and the whole contents of these tubes, the products of absorption, are finally poured into the venous blood at the base of the left side of the neck. The function of this lymphatic system is that of taking up and returning to the blood the surplus fluids which have escaped from the vessels for the nourishment of the various parts of the body. Also in connection with the organs of digestion, under the name of lacteals, by similar vessels, many of the foods, altered in appropriate ways, are absorbed and conveyed into the blood. From this short explanation you will see what an arrangement exists in the body both for good and evil. By means of it not only are foods and the surplus of nourishment to various parts of the body conveyed into the general stock of blood, but also the benign products of inflammation; and organs which it is desirable in the natural course of life should dwindle, as being no longer required, are got rid of by being converted into fat, which is then absorbed into the blood, and finally conveyed out of the system by one or more of its excretory channels. This is the good side of the picture; now we come to the bad one. For the same system absorbs, as I have said, noxious products, and poisons the blood and system generally with them. You will often see this taking place. A patient will be admitted into the hospital with, let us say, a wound on the hand, and you will be told that the wound has "festered." You will see the surgeon not only examine the inflamed part, but the whole of the arm right up to the arm-pit. You will see him examine the skin of the fore-arm and arm, and you will hear him make inquiries as to whether there is pain, especially at the bend of the elbow and at the armpit. If the case is at all severe you will probably see a thin red blush running up the arm, and you will find one or more painful lumps at the flexures of the joints; this thin red blush marks the course of a lymphatic vessel which is inflamed from carrying poisonous fluids, and the lumps are enlarged and painful glands, irritated by the same agents. The various disease-spreading agents have their various effects here as elsewhere. Some act so irritatingly that suppuration and abscess in the glands speedily result; others pass through this system, doing but little damage there, and exercise their full effect on the body generally through the medium of the blood; whilst others, again, start a fresh life in the glands which they have diseased, and, whilst working their mischief here, still manage to reach the blood, and so infect other parts at a wide distance from whence they started. Where inflammation occurs one result, fever, is pretty constant. The word fever is derived from the Latin one, Ferveo, to be hot, and one main symptom of the condition is a rise of the temperature of the body. To see its full effects we should observe the various systems, noting, one by one, the changes which fever effects in the natural workings of the same. Thus, in the circulatory we have (when the fever is of the ordinary type) a rapid, strong action of the heart and corresponding quickness of the pulse; in the nervous we have headache, confusion of thoughts, extra sensitiveness to light and sound, and want of sleep; in the digestive, loss of appetite, thirst, constipation; and in the excretory, lessened discharge of urine, and heat and dryness of the skin, with lessened perspiration. These general symptoms—greater heat of the body than is natural, headache, dryness of skin, loss of appetite, constipation—are not all constant in fevers: some of them are sure to be present, but some of them may not only be absent, but may be replaced by their very opposites, in this way marking the variety of fever with which we shall have to deal. The one almost constantly present is increased heat, and we will accordingly deal with that first. The normal, or natural, mean average of body temperature, as measured by the clinical thermometer, is 98·4 degrees Fahrenheit. I say the average advisedly, for it ranges a little during the twenty-four hours, being a little higher than what I have stated between four and nine p.m., and lower between half-past one and half-past seven a.m. When fever is prevailing it is said to be of a certain type, according to the variations which are found in the body-temperature on using the thermometer. Thus, if the temperature is always above the normal point, and day after day the thermometer shows almost the same rise and fall, by evening and morning observations, we are dealing with a continuous fever; if fever prevails continuously but in such a manner that sometimes temperature is much lower than at others, we have a remittent fever; and if temperature, after rising, has fallen quite to the normal point, and remains there for a longer or shorter time, and then again mounts above the normal line, the fever is said to be of an intermittent type. I must direct your attention to another point of view of temperatures in fever, and that is to what they signify. If the record be up to 101 degrees fever is spoken of as being slight; it is moderate when 103 degrees has been attained; 104·5 degrees is said to be high; hyperpyrexia, or over feverishness, is said to have been reached when 106 degrees is registered, and then we are dealing with a most dangerous condition in which the heat engendered in the body, if it persists, kills by destroying the elemental life of the tissues; and few have survived temperatures of 107 or 108 degrees. Observe, then, that with the thermometer you watch both the type of fever which is prevailing and the range of temperature.

### Surgery at Home.

Whilst some women are undoubtedly born nurses there are but few born surgeons. Nevertheless the wife of a farmer named Merritt, of Dubbo, New South Wales, must be reckoned amongst the latter. John Merritt cut his throat with a razor, and just missed the windpipe and carotid artery. His better half immediately stitched up the wound with a packing needle and twine, and then the constable marched the invalid off to prison.

### Post-Graduate Clinics for Nurses.

By a Trained Nurse.

### XXVII.—SICK DIET AND COOKERY.

The complexities of modern nursing are admirably illustrated not only by the evolution of elaborate surgical dressings and the multiplication of sick-room appliances, but also by the science which is now included in the dieting of our sick.

Looking back, it does not seem to be many years since the sick person unable to take solid food was dieted on substances known generally as "slops." The translation of this term was delightfully simple. For the edict being medically pronounced, the invalid forthwith was fed on gruel, toast-water, broth, and beef-tea—of a kind.

Now, even if beef tea be well made it must prove monotonous as a steady diet, and calves'-foot jelly, the old standing dish of centuries of sick-rooms, however redolent of lemons and sherry, must have been taken on sufferance when it made so regular and unvaried an appearance at the bedside. Myriads of patients have recovered in spite of such primitive methods of sick diet. But doubtless many thousands were sacrificed to the starvation entailed by the beef-tea and jelly *régime*. We have changed all this, and a hundred and one niceties and chemical subtleties have been added to dietetic resource. But we have not yet reached that desirable stage of progress which will enforce a sound and practical training in cookery and sick diet before the nurse is considered worthy of the honour of placing "trained" before her appellation. At present we have not entirely freed ourselves from a lingering suspicion that the art of cooking is in some sense derogatory to dignity. But nurses are a progressive body of women, and a large percentage of them are beginning to realise that it may be just as dignified—and it is certainly more difficult—to compound the ingredients of a successful sick food as it is to compound the drugs which found a successful prescription. Dr. Johnson by no means considered cookery beneath his dignity, and used to boast he could write a better cookery book than had ever been written. And he never forgot to add somewhat contemptuously, "A woman may spin, but she cannot make a good cookery book," in which sentiment everybody may not agree. I cannot help maintaining that no nurse is thoroughly equipped for private work who is not conversant with the resources of the saucepan. A love of cookery is essential to successful nursing, and no nurse should rest satisfied till she can herself cook any dish likely to be set before a sick person. She should be fertile of resource as to sick-diet, and a walking encyclopædia of culinary suggestion. The first axiom applied to dietary is that food taken with enjoyment is more readily digested than is food taken on sufferance. And, moreover, *l'appetit vient en mangeant* is the truest of French proverbs. But, of course, it goes without saying that "the appetite" which comes through eating comes only when the food offered is well-flavoured and palatable, presented in a form pleasing to the eye, and suited to the patient's needs. The meal enjoyed and well digested gives energy to the nervous system, making both appetite and digestive power for the next meal; while, on the other hand, monotonous feeding and food taken for duty's sake is too often only the last straw which breaks the back of the patient's appetite. A knowledge of sick-diet belongs to the ambition of the individual nurse. For, so far, no one training school has identified itself particularly with this branch of training. So that the nurse possessed of skill in cookery and special knowledge of sick-diet has acquired it by her own exertions; and this is the more to her credit, since it is knowledge acquired with difficulty. It would be impossible for the nurse electing to do private nursing to exert herself in a more valuable direction than in learning all she can on this important subject. For the average cook of the average household has a most limited knowledge of the needs of the sick, and in the early stages of an illness, perhaps before a trained nurse has been requisitioned, the invalid has endured a surfeit of boiled soles, bits of chicken, and ineffectual beef-tea—the beef-tea which has become so sickly to him and brings back the recollection of weary, feverish nights and painful days. The first sight and well-remembered taste takes all further appetite away, and though his system be craving food, he feels that he needs it not, simply because he has no desire for the particular kind of food brought to him. And so convalescence is retarded, and a patient sorely lags in picking up health and strength, all for the want of the right food given at the right time.

I would have every nurse an artist in food, and I would like her to feel that the arrangement of an invalid's tray—its decoration and its setting forth—and the contents of his feeding cup are as worthy of her talent as is the canvas of the hand of the great artist. For the recovery of a sick person to health and strength through the medium of dainty, well-chosen foods is, in very truth, a triumph of art in cookery and nursing. It seems to me it is as much bad nursing to set food before her patient which is not palatable and pleasing as if the nurse were to put on a poultice too hot or to fill a water-bed with cold water. The sooner we get rid of the idea that the cook is responsible for a sick person's food the better. Of course, in some rich households the cook will prepare excellent chicken broths and teas. But we are dealing with the average of things. And the average cook has no conception of the chemistry of invalid foods. It is beyond her, and the nurse must make it her province.

The patient's friends will be able to give information as to his "fads" about food—what he likes and dislikes—the number of sugar lumps he takes with his tea, whether he can take coffee and cocoa, and a hundred other little hints. It annoys a sick person to find his nurse unacquainted with his "ways," and it is very trying for him to have the responsibility of initiating the nurse into his dietetic eccentricities. In my sick-diet clinics it is very improbable that I shall so much as mention hydro-carbons or carbo-hydrates, as such. For I wish to make my sick-diet chapters eminently practical and helpful. And while I would have every nurse conversant with chemical food-values, I am fully aware that such knowledge does not prevent a nurse from serving up burnt beef-tea and mawkish soft toast to her sick patient. I have seen a Gold Medal nurse give her patients cod-liver oil five minutes after a meal and administer mutton broth with half an inch of fat floating on the top. "I have skimmed it," she said, "but it won't all come off." Of course, it would not, for although she understood all about carbo-hydrates she had no practical culinary knowledge as to how mutton broth might be relieved of superfluous fat. During a serious illness I was attended by two nurses from the same training school. One of them served my diet faultlessly. Another invariably "smoked" the broths when heating them. And she seemed constitutionally unable to prepare cocoa without burning the milk of which it was composed. She was "trained" from the average standpoint. But I would have prescribed three months' service in a diet kitchen. The kind of food the patient may take is entirely a medical question. The variations, mode and method of administering, belong to the nursing department.

### Jubilee in the African Karoo.

By a Correspondent.

The remotest spots in Her Majesty's dominions made some effort to commemorate her Diamond Jubilee; and I think not many can be more remote than the one in which I spent mine.

At about nine o'clock on the morning of June 21st, 1897, we started, a party of eight persons, in a waggon drawn by twelve oxen, for a sixteen-mile ride to the little Dutch village of Steytterville, situate in a South African Karoo. When I say eight persons I forget the four Kaffir servants, two male and two female, who made up our number to twelve. On our road we were joined by another waggon, containing two old ladies, neighbours of ours, their guest, a boy about seventeen, a Dutchman, and four Kaffir servants also. Both waggons were well provided with beds and provisions for three days, for travellers not only travel in their waggons, they also live in them. They are not unlike a gipsy van.

About two o'clock p.m. a halt was made, the oxen were outspanned and sent to feed, and we proceeded to feed ourselves. The servants quickly made a fire and a big kettle of coffee, chops were fried on the gridiron, thick slices of bread and butter were cut, and enjoyed as such meals are when spending a glorious day in the open air. Dinner ended, the oxen were spanned in again, and on we went, with that lazy, leisurely progress characteristic of oxen, at a speed of about three miles an hour, along a road bare, sandy, and uninteresting.

Steytterville was reached at about half-past six p.m., and as these are our short winter days, it was quite dark. Five of the party were going to live in the waggon all the time, but three of us, of whom I was one, stayed with friends, a most hospitable Dutch family. The mother, an extremely kind-hearted old lady, was also one of the most voluble persons I have ever come across; and, strangely enough, out of her large family, three—a daughter and two sons—are deaf and dumb. They have not inherited it from her, that is certain.

Jubilee Day dawned brightly enough; but, alas! up came a regular South African gale, and with it a regular South African dust-storm. Clouds and clouds of fine sand obscured the horizon, got into one's eyes, ears, and mouth, stung one's face, and made all enjoyment, to say the least, difficult. However, the programme of the day was conscientiously carried out; the volunteers marched past, the children—a large number—received their medals commemorative of the day, dinners were given, sweets scrambled for, and the usual sports took place—all under difficulties. The pig was caught by his greased tail, the donkeys refused to race, the three-legged race levelled the usual number in the dust, and so forth.

Towards evening the gale increased, if possible, and the Chinese lanterns and the fireworks suffered wofully. Nevertheless, crowds were out to see them, and managed to enjoy themselves more or less. There were a great number of Dutch people in the village—the majority, in fact, were of that nationality—their waggons were spanned out here, there, and everywhere; and it is satisfactory to note that there were no dissentient voices among them, but all expressed hearty loyalty. As for the natives, they had a spree, and that was quite enough to ensure their loyalty.

At this little out-of-the-world post office the Queen's gracious message was received just two minutes after its despatch from the centre of civilisation.

The next morning the gale still continued, but with less violence, and it was possible to take a drive out to a new road which is being made, at the cost of much labour and much dynamite, along the side of the mountain and above the Great River. On the morning of the previous day the chief engineer of this road fired off 21 successive charges of dynamite, and then assembled all the workmen—over a hundred in number—to sing "God Save the Queen" and receive a day's pay without working for it. We were rewarded for our drive by a very fine view, for we were now among the hills, where there is some really grand scenery. On our return, however, the wind and the dust were in our faces—very much so.

Of course this was a grand opportunity for the interchange of visits between friends who, perhaps, live far apart and seldom meet, except on some occasion such as this, which brings them all together. There was, therefore, a great deal of meeting and greeting and running from house to house. Moreover, on Wednesday morning all the stores were open for a few hours and drove a roaring trade with the farmers and their wives in from the surrounding country. "Surrounding country" here means any place within 20 or 30 miles.

Meanwhile, our oxen have been sent for; they have spent their Jubilee feeding at a neighbouring farm; they do not turn up till four in the afternoon, and it is five by the time we start homewards. At first we walk to the top of the hill, where we turn and look back upon the little village, characteristically Dutch, with its wide straight street about a mile long, planted with rows of little trees on either side; then, as the short day closes in and the air grows chilly, we are glad to climb into the waggon and sit or lie on the beds looking out at the darkening sky and the wide solitary landscape.

At nine o'clock we spanned out for supper. The night was cloudy, dark, and still, the wind having gone down, and it was not at all cold. The wood fire soon blazed up merrily, and we all formed a circle round it, the Kaffir servants cooking the meat and making the coffee, while we chatted and told stories. The two waggons stood side by side, and the oxen being tied fast lay down quietly on the ground. The firelight threw a weird glow over everything, forming a picturesque and most characteristic South African scene.

And now we spanned in again, and the slow jolting journey continues through the dark still hours. We all lie down, packed rather like herrings, some fall sound asleep, others find sleep impossible; the weird cries and ejaculations of the drivers encouraging their oxen and the creaking and jolting of the waggons are the only sounds that break the silence.

Now, here we are at the little solitary farm which is our home. Doors are never locked here, so we have only to steal quietly in and turn into bed as quickly as may be. It is half-past one a.m. on the morning of Thursday, June 24th—and our Jubilee is over.

### American National Red Cross Society.

The headquarters of this society are in Washington. Miss Clara Barton is the president, and bears all the expenses of the establishment out of her private fortune. The house was at one time the headquarters of General Grant during the Civil War, and it is now marked by a white flag bearing the red cross. Inside the walls are covered with the flags of many nations, the red flag of Switzerland occupying the place of honour. A corps of nurses and physicians were sent to Cuba, whilst Miss Barton and others have been working during the late War in Turkey. The services of the American Red Cross have been widely appreciated, to which a casket of badges and jewels, gifts from royal persons and societies, standing in the president's room at Washington, bear testimony.

### The Etiquette of Nursing: A Reply.

By MARY GARDNER, Matron City of Norwich Isolation Hospital.

I HAVE read with regret the article on "The Etiquette of Nursing," by G. A. Hawkins-Ambler, F.R.C.S. We have lately had much unfriendly criticism of nurses by medical men, and it is not a hopeful or encouraging sign to find these symptoms of suspicion and distrust between two professions so closely allied and mutually dependent that it may almost be said of them,

"Useless each without the other."

The comparatively recent articles in a medical journal, the *Practitioner*, descended to a level of abuse which made them beneath notice. The present essay, although far less offensive in tone, is yet too sweeping in condemnation and does grave injustice to nurses as a class. I assume the writer does not intend to be taken too literally, and I therefore pass over obvious and half-humorous exaggerations. But when all allowance has been made the charge resolves into one of want of honour and loyalty in the nursing profession as a whole. He practically asserts that he has never met a nurse who recognised the duty of loyalty and obedience to the doctor under whom she works. We must conclude that he has never done so by the sacrifice which he says he is prepared to make for such a nurse could she be found! His experience of nurses, judging from the examples quoted and the general tone of his article, would appear to have been singularly unfortunate. My own, extending over eleven years, has been on the whole very different. Although I do not deny that there are some who through thoughtlessness, ignorance, or worse, the lack of honourable instinct, act in the manner he has described, my sincere belief is that they are exceptions to the rule.

Mr. Hawkins-Ambler has, perhaps unconsciously, indicated the cause of much difficulty where he says, "A nurse is usually trained on one person's fad, and takes the fad for science, seizing with a woman's thoughtlessness and enthusiasm on the unnecessary and worthless in place of the abiding and true. She is at some pains to grasp the lesson she is taught, and she holds the teacher the one authority, his method the only one." There is truth in this, but is it not the fault of the faddist? Who is generally to blame if the inexperienced nurse thinks the treatment she has been taught is the only right one? Are doctors always sufficiently liberal and broadminded to acknowledge the merits of other men and other methods? Are they loyal to each other? I have heard from medical men expressions of the utmost contempt for the opinions and practice of those who differed from them. I have been compelled by professional etiquette to listen in pained and silent disapproval to condemnation and even ridicule of the very methods in which I had been trained by teachers whom I held in the highest esteem. This is the reverse side of the shield. Those who sin thus against professional loyalty and good taste are usually the younger men, newly qualified and flushed with the confidence of inexperience, but it is not always so. I have met men of high standing and reputation who were very intolerant. In the early days of my nursing career I nursed a private case for an eminent London physician, who condemned in unqualified terms a mode of treatment then much used by other men, equally successful with himself, saying that he could not find words strong enough to express his abhorrence of the practice. This might have had an injurious effect upon many young nurses, but happily for me I had been trained under a matron who was as wise as she was clever, who, while always impressing upon her pupils the supreme duty of loyal obedience, prepared us for encounter with many men, many opinions. She gave us in clear and simple style some insight into the principles underlying various modes of treatment, showing us the relation of different methods leading to the same result. The idea thus gained of unity of aim in variety of method has been of infinite service to me since in helping me to an intelligent appreciation of my work as a nurse. None could teach this lesson to nurses so well as could doctors, but do they? Rarely, if ever, I think. There is too great a tendency to exclusiveness in teaching. While the doctor has an unquestionable right to demand that his own mode of treatment shall be faithfully and rigidly carried out without a suggestion of doubt or demur, he is not himself fulfilling the golden rule if either expressly or by implication he teaches the nurse to despise the practice of other men. It is, however, I should say, almost impossible for any experienced nurse, especially in private work, to continue a fanatic with regard to any treatment. Coming in contact, as she inevitably must, with so much variety of opinion and practice, if she does not fall into the opposite error of becoming a medical sceptic, she recognises in time that every established mode of treatment can show good results, that none is infallible, and none can be of universal application. Above all, that the success of any depends upon the loyal co-operation of the nurse, and, therefore, even if unable to accept each in turn as "wisest, virtuousest, discreetest, best," she will, if a true woman and well-trained nurse, do her own part to the best of her power, keeping within the limits of her responsibility. The nurse who thinks everything wrong which is not done according to the ways of the hospital in which she was trained has her counterpart in the medical student or recently-qualified medical man whose absolute belief in the superiority of the methods of his school cannot be surpassed, and who looks down on the older generation, but neither is typical of the profession to which they belong.

In conclusion, I also would welcome the introduction of a more definite and systematic code of ethics among nurses. At present, conduct depends upon individual character and training, and the result is variable; yet I believe that the great principles of loyalty and obedience which are the very foundation of our work are far more generally acted upon than Mr. Hawkins-Ambler represents. One thing I have never found, *i.e.*, a nurse writing or speaking against doctors as a class. In this the loyalty of nurses is apparent. Even though in defence of my order I have been compelled to hold some medical men answerable in a degree for the very faults of which they complain, I do not disparage the profession as a whole, nor will ever do so, for I freely acknowledge that nurses have no better friends than doctors. Personally, I have every cause for gratitude and esteem, and yield to none in sincere admiration and respect for the medical profession, of which it was said more than two thousand years ago, "Honour the physician with the honour due unto him . . . for of the Most High cometh healing, and he shall receive honour of the King."

### Minor Appointments.

SHEFFIELD UNION INFIRMARY.—Miss Rosina M. P. Rochford was appointed on August 18th Night Superintendent of this infirmary. She received her training, and afterwards held the posts of charge nurse and night superintendent, in this same institution. Miss Jane C. Gronow was appointed Maternity Nurse on the same date. She received her general training at the Royal Infirmary, and her midwifery training at the Ladies' Lying-in Hospital, Liverpool. Miss Gronow has been charge nurse, Liverpool Royal Infirmary; head nurse, Bury Dispensary Hospital; district midwife to Liverpool Ladies' Charity and Lying-in Hospital; and charge night nurse at Hunslet Union Infirmary.

SEACOMBE, CHESHIRE.—Miss Jessie M. Cawley was appointed Charge Nurse of the Cottage Hospital on August 24th. She was trained at the Northern Hospital, Liverpool, and received her midwifery training at the Manchester Maternity Hospital. Miss Cawley holds the L.O.S. diploma.

THE NEW INFIRMARY, LEWISHAM.—Miss Jessie Fraser Ballantyne was appointed Sister of this institution on August 16th. She was trained at Guy's Hospital, and was staff nurse of the same hospital from 1894 to 1897.

### A New Convalescent Home.

From a Correspondent.

Down under the Castle Hill at Bletchingley Mr. Partridge has built a beautiful little convalescent home to the memory of his daughter. The home has been open for a month, but the eight beds are not yet full, which means that the home should be more widely known. Miss Longbottom, the matron, is a Westminster nurse, and was at one time home sister at the London. She is thoroughly competent and the home is thoroughly comfortable, and this is necessary, for the class of patients is to be "superior." When Mr. Partridge built and furnished the home he was thinking of the needs of poor teachers, clerks, and shop assistants—people to whom the ordinary convalescent home, with its many restrictions and mixed society would not be restful—and he decided that his own pretty little building should be reserved for these. Men and women are taken, and there is absolutely no charge. It may interest readers of The Hospital to know that the first patient was a tired, overworked nurse. Patients are admitted from London or other hospitals, or direct from the physician in charge of the case, and all applications should be addressed to the Matron, Convalescent Home, Castle Hill, Bletchingley, Surrey. The home faces south, and looks right across the Weald of Sussex and out to the Isle of Wight; it is sheltered to the north and east by the Castle Hill; the soil is sandy. It is an exceedingly favourable site, high and breezy and yet dry and warm. Rheumatic cases especially should do well there. All the appointments are hygienic and yet artistic; there is a splendid piano, a nice collection of books, pretty bed tables, a box of games, &c. Certainly the home should prove of the utmost benefit to those for whom it was designed.

### Appointments.

### MATRONS.

Shoreditch Infirmary.—On July 28th Miss Emily Jardine was appointed Superintendent Nurse of the above infirmary. She was trained for two years at the Kent and Canterbury Hospital, and has since spent seven and a half years in India. She was in the Indian Nursing Service for five years, and for the last two of these she held the post of sister. The rest of the time she was engaged in private work.

Retford Dispensary and Cottage Hospital.—On August 18th Miss Sophia F. Coulthurst was appointed Nurse Matron of the above hospital. She was trained at the Liverpool Royal Infirmary Training School, and became afterwards charge nurse and night superintendent of that infirmary. Subsequently she was district nurse at Bebington, Cheshire, and then night superintendent at the Royal Infirmary, Hull.

General Hospital, Birmingham.—On August 19th Miss Harriett A. Marriott was appointed Matron of this hospital. She was trained at St. Bartholomew's Hospital (1890-3), and subsequently became staff nurse there for a year, after which she held the post of sister at the General Hospital, Birmingham, for three years. Miss Marriott was selected out of 84 applicants for her present appointment.

Memorial Hospital, Jarrow-on-Tyne.—Miss Neech was on Wednesday, August 18th, appointed Matron of the Memorial Hospital, Jarrow-on-Tyne. Miss Neech received her training at the Royal Infirmary, Glasgow, where during the past two years she has held the position of charge nurse.

Kensington Infirmary.—Miss Rebecca Evans was appointed, on August 17th, Sister at the above. She was trained at Ladywell, Eccles, near Manchester, and at Salford Union Infirmary, Pendleton, Manchester, where she also held the post of charge nurse. She has since held the post of charge nurse at Brook Hospital, Shooter's Hill, London, S.E.

### Everybody's Opinion.

[Correspondence on all subjects is invited, but we cannot in any way be responsible for the opinions expressed by our correspondents. No communication can be entertained if the name and address of the correspondent is not given, or unless one side of the paper only is written on.]

### THE ROYAL BRITISH NURSES' ASSOCIATION.

Nurse Hephzibar Walker writes: As an old member of the R.B.N.A. I went to the annual meeting last month, and, like many others, came away with a feeling that it was a disgrace to the nursing world without having the advantage of being a help to any one. This was, perhaps, due partly to the fact that very many of those present knew little or nothing of the matters in dispute, and I would suggest to those members whom I heard complaining that they did not understand the working of the Association, that they should write to the secretary (whom I have always found most courteous and willing to help) for a copy of the bye-laws, or that they should take an opportunity of seeing her personally, and getting an explanation of those things they don't understand. We members wish to be treated as sensible women; let us behave as such and use our own experience and common-sense instead of allowing ourselves to be influenced by those who talk the most and make the most noise. Each member has a responsibility of her own and, if we are to show our motto, not only on our badges but in our membership, let us all take an intelligent interest in the Association, which was started entirely for the welfare and advancement of nurses and those connected with nursing work. If this be done our annual meetings will begin to show a record of work instead of noise, and things will be soon in a fair way towards satisfactory working. And above all, let nothing be done which shall seem to outsiders like disrespect to the doctors, who are after all not only our superior officers but also our best friends.

### POINTS IN SICK NURSING.

Letitia Lloyd writes: I trust I shall not be thought captious if I venture to remark on one or two points about the management of a sick-room, in which, according to my experience, even the best trained nurses often fail. No one can better appreciate the good sick nurse of the present day or feel more grateful than myself for the help she gives in times of illness, but the little carelessness that I would mention is so easily avoided, and though apparently trifling is so often productive of discomfort to patients and their friends that I think it better to ventilate the grievance. And first, why will not a nurse cleanse the drinking vessels in the sick-room directly after use? I have often come on duty with invalid friends to find remains of beef tea, milk, &c., in the feeding cup, and dregs in the medicine glass, which neglect both has a slovenly appearance, and causes delay if the patient happens to want a drink of anything quickly. I find it myself more expeditious, as well as better, to at once rinse out any glasses, cups, &c., when used, and dry them with a clean glass cloth that can be always kept at hand; they are then clean and ready when wanted again. My second indictment is that nurses are often regardless of the temperature of a sick-room, not, of course, in those cases where it is of vital importance, but still where it may make a decided difference in the comfort and well-being of the patient. I have known the thermometer in a sick person's bed-room vary quite ten degrees in the twenty-four hours (from positive chilliness to real heat, that is), and when I remarked on it to the nurse, her answer was, "Oh, the doctor said *about* sixty degrees, you know," her interpretation of *about* being from five degrees below to five degrees above the indicated point. Another time a patient complained to me that he constantly got so hot and oppressed through the evening that he could not sleep till very late, and then woke early in the morning, feeling quite as uncomfortable from cold. The explanation was simply this—that the nurse having kept a moderate fire all day—it was in early spring—so that the room was perhaps a little below sixty degrees, began systematically to pile it up when the house was shut and lighted up for the evening, with the result that by ten p.m. the temperature had risen to over sixty-five degrees, and the place with fire and lamps felt close as well as hot. She then went to bed. Her patient not being very ill, she slept through most of the night, or at least did not rouse herself to attend to the fire, which by about five a.m. was of course almost out; and in the most chilly time of the morning the temperature had gone down below fifty-five. No wonder the invalid suffered from extremes of heat and cold. I think that nurses should be additionally careful of all details connected with the sick-room now that doctors leave these so much more in their hands than was the case in former days when skilled attendance was rare.

### For Reading to the Sick.

"FEAR NOT."

**Verses.**

O could we make our doubts remove
The gloomy doubts that rise,
And see the Canaan that we love,
With unbeclouded eyes!

Could we but climb where Moses stood,
And view the landscape o'er;
Not Jordan's stream, nor death's cold flood,
Should fright us from the shore.
—*Isaac Watts.*

Mine is an unchanging love,
Higher than the heights above,
Deeper than the depths beneath,
True and faithful, strong as death.

Thou shalt see My glory soon,
When the work of grace is done;
Partner of My throne shalt be—
Say, poor sinner, lov'st thou Me?
—*W. Cowper.*

Man may trouble and distress me,
'Twill but drive me to Thy breast;
Life with trials hard may press me,
Heaven will bring me sweeter rest.
O 'tis not in grief to harm me,
While Thy love is left to me.
O 'twere not in joy to charm me
Were that joy unmixed with Thee.
—*H. F. Lyte.*

**Reading.**

Fear not, little flock, it is your Father's good pleasure to give you the Kingdom.—*Luke* xii. 32.

The little flock. The music of the Shepherd's voice again! another comforting word, and how tender. His flock, a little flock, a feeble flock, loved of the Father, enjoying His "good pleasure," and soon to be a glorified flock, safe in the fold, secure within the Kingdom! How does He quiet their fears and misgivings? As they stand panting on the bleak mountain side He points His crook upwards to the bright and shining gates of glory and says, "It is your Father's good pleasure to give you these!" What gentle words! What a blessed consummation!

"It is your Father's good pleasure." The Good Shepherd, in leading you across the intervening mountains, shows you memorials of paternal grace studding all the way. He may "lead you about" in your way thither. He led the children of Israel of old out of Egypt to their promised Kingdom—how? By forty years' wilderness, discipline, and privations. But trust Him; dishonour Him not with guilty doubts and fears. Look not back on your dark stumbling paths; nor within on your fitful and vacillating heart; but forwards to the land that is far off. Let the melody of the Shepherd's reed fall gently on your ear. Fear not, little flock, though yours for a while should be the bleak mountain and sterile waste, seeking your way Zionward, it may be with torn fleeces and bleeding feet, for "it is not the will of your Father which is in Heaven that one of these little ones should perish."

### Notes and Queries.

**The contents of the Editor's Letter-box have now reached such unwieldy proportions that it has become necessary to establish a hard and fast rule regarding Answers to Correspondents. In future, all questions requiring replies will continue to be answered in this column without any fee. If an answer is required by letter, a fee of half-a-crown must be enclosed with the note containing the enquiry. We are always pleased to help our numerous correspondents to the fullest extent, and we can trust them to sympathise in the overwhelming amount of writing which makes the new rules a necessity. Every communication must be accompanied by the writer's name and address, otherwise it will receive no attention.**

*A Twenty Year Old Medical Scandal.*

(354) I am anxious to obtain accounts of a home for incurables or invalids in the southern counties managed by a noted doctor in the neighbourhood of Dorset or Devonshire, which was closed by a Government order some twenty years ago.—*Enquirer.*

Can any of our readers help?

*How to Enter Army Nursing Service.*

(355) Will you kindly inform me what are the qualifications necessary to become an army nurse? (2) and what are the best steps to take in order to do so?—*Catherine.*

(1) The qualifications are three years' training and service in an approved civil general hospital, where "adult male patients receive medical and surgical treatment"; the age to be between 25 and 35; and a recommendation from a person of good social position that they are desirable persons to enter a service composed of ladies. (2) Send for forms of application to the Adjutant-General of the War Office, Pall Mall, S.W.

*Paying Patient at Medicinal Bath.*

(356) Can you tell me of any hospital or home where a patient could be admitted on small weekly payment to undergo mineral water treatment for rheumatism. I find that the hospitals for the treatment at Bath, Buxton, and Harrogate only admit patients too poor to pay anything.—*Nurse Agnes.*

There are convalescent homes which would receive your patient on the terms you wish if she is able to walk, at Buxton, Bath, &c., of which we can give addresses. See answer to query No. 353, the information in which is given by a correspondent from personal experience.

*Nursing at St. Thomas's.*

(357) Would you kindly tell me what kind of hospital St. Thomas's is? (2) At what age probationers are taken? (3) The price of Honnor Morten's book?—*Crouch End.*

St. Thomas's is one of the oldest and largest hospitals in the metropolis. (2) Probationers, paying and non-paying, are trained in connection with this hospital by the "Nightingale School," age 24-30. (3) The price of H. Morten's book is 2s. 6d.

*Weakness of the Spine.*

(358) Will you kindly tell me of a free institution where I could have a course of massage treatment for weakness of the spine?—*F. F. Clay.*

Write to the Secretary, The National Hospital for the Paralysed and Epileptic, Queen's Square, Bloomsbury, W.C., and state your case.

*Where Can Nurses Disinfect?*

(359) Will you kindly tell me whether there are any homes or places within a few miles of London where nurses can disinfect after fever cases, and where terms are moderate?—*F. B.*

The Mary Wardell Convalescent Home for Scarlet Fever, Stanmore, Essex. Terms, women, 15s. a week; private patients, £3 3s. Miss Wood, The Nurses' Hostel, Percy Street, makes arrangements for nurses to disinfect.

*As Children's Nurse Abroad.*

(360) Will you kindly give me address of registry office through which I could get a situation as children's nurse abroad.—*Peggy Pierce.*

It is against our rules to give such an address. The best way to find what you want is to see advertisements in *Morning Post*, and failing that to advertise. You must make full inquiries before taking a post, as young girls are exposed to great dangers abroad.

*Monthly Nursing.*

(361) Will you kindly tell me if there is a lying-in hospital in Liverpool where a paying probationer can obtain a certificate after a few weeks' training?—*Certificate.*

Liverpool Ladies' Charity and Lying-in Hospital, Brownlow Hill.

*Assistant Nurses.*

(362) Can you tell me of any general hospital (not infirmary) in London or near, where assistant nurses are taken?—*H. C.*

It is necessary to send name and address, as a pledge of good faith, before we answer questions.

*Dispensing.*

(363) I should be glad if you will tell me what subjects I should have to take up in the examinations which qualify for dispensing. (2) Should I, in the ordinary course of things, be able to take a post as assistant dispenser at the end of the first year whilst continuing my study at the hospital? (3) Is the six months' course any good, or would you recommend my taking the two years' course? (4) Is there a good opening for qualified dispensers, and what is the salary paid?—*Apothecary.*

(1) Write to the Secretary, Apothecaries' Hall, Blackfriars, E.C.; or to the Secretary of the Pharmaceutical Society of Great Britain, Bloomsbury Square, W.C., for information. (2 and 3) The subject is fully gone into in "Burdett's Annual" for 1894. (4) There is an opening for women dispensers.

*Nursing in a Bracing Climate.*

(364) *A. E. M.* is reminded that our rules require name and address of our correspondents as a pledge of good faith.

*Wanted an Address.*

Can any of our readers give us the address of Nurse Dyer, late of Sale Street, Paddington? If Nurse Dyer will apply to the Editor, she will hear something to her advantage.

